# Construction of a prediction model for sarcopenic obesity based on machine learning

**DOI:** 10.3389/fpubh.2025.1576338

**Published:** 2025-06-27

**Authors:** Mengru Xu, Jia Liu, Song Hu, Tongxiao Luan, Yuting Duan, Aohua Wang, Ziwei Cui, Jing Zhou, Yongjun Mao

**Affiliations:** ^1^Department of Health Care/Geriatrics, Affiliated Hospital of Qingdao University, Qingdao, Shandong Province, China; ^2^Qingdao University, Qingdao, Shandong Province, China

**Keywords:** sarcopenia, RF, calf circumference, obesity, machine learning

## Abstract

**Background:**

In the context of the rapidly aging global population, sarcopenic obesity (SO) in older adults is associated with significantly higher rates of disability and mortality. SO has become a serious and critical public health concern. This study aimed to develop and validate predictive models using machine learning (ML) to identify SO in patients.

**Methods:**

Data from 386 participants collected at the Affiliated Hospital of Qingdao University were divided into an 8:2 ratio, with 80% used for training and 20% for testing. Univariate analysis was performed to identify the factors correlated with SO, and multivariate logistic regression analysis was performed to determine the independent factors influencing SO. The Shapley Additive exPlanations (SHAP) diagram was used to illustrate the importance of variables in the model. To develop a predictive model for SO, we used five models and applied internal five-fold cross-validation to determine the most suitable hyperparameters for the model.

**Results:**

Among 386 participants, 61 were diagnosed with sarcopenic obesity (15.8%). We identified four independent predictive factors, namely BMI, Barthel Index score, grip strength, and calf circumference. Notably, calf circumference plays an important role in assessing the risk of SO in older adults. The area under the curve (AUC) values of the test set for the Random forest (RF), naive Bayes (NB), Light Gradient Boosting Machine (LightGBM), k-nearest neighbor algorithm (KNN), and eXtreme Gradient Boosting (XGBoost) models were recorded as 0.839, 0.815, 0.808, 0.794, and 0.798, respectively. Among these models, the RF model exhibited the best average performance in the training set, with an AUC value of 0.839.

**Conclusion:**

We constructed a predictive model based on the results of the RF model, combining four clinical predictors—BMI, Barthel Index score, grip strength, and calf circumference—to reliably predict SO.

## Introduction

Sarcopenic obesity (SO) is a clinical condition characterized by high body fat and low muscle mass (1). While this condition can affect individuals of any age, it is more prevalent in older adults. Studies have shown that muscle mass decreases at a rate of 0.5 to 1.0% per year after the age of 30 (2). This condition leads to a reduction in skeletal muscle mass and strength, an increase in fat mass, and a redistribution of body fat. This significantly increases the risk of physical decline, reduces quality of life, and raises the rates of disability and mortality in older adults (3–6). One of the physiological reasons for the pathological effects of obesity on skeletal muscle function and quality is its pro-inflammatory nature. The accumulation of excess fat (systemic, truncal, or visceral) stimulates the immune system to produce higher levels of inflammatory cytokines, such as interleukin-6, tumor necrosis factor-alpha, and C-reactive protein (7–9). This increased production of inflammatory markers, in conjunction with muscle growth-inhibiting factors, ultimately leads to the loss of muscle mass and function (10). Obesity can also exacerbate muscle atrophy by increasing fat infiltration into muscles, further reducing physical function and increasing the risk of death (11). The development of sarcopenia can exacerbate glucose abnormalities and insulin resistance associated with obesity (12), both of which intensify metabolic disorders in the body through their synergistic effects, increasing the incidence and mortality (13). Compared to individuals with only sarcopenia or obesity, those with sarcopenic obesity have a higher prevalence of adverse health outcomes and an increased risk of metabolic syndrome, along with a more serious medical burden and social pressure. Due to the slow progression of sarcopenic obesity, insufficient attention and intervention in the early stages of the disease lead to delays in diagnosis. These delays can have significant consequences for quality of life and all-cause mortality (14–16). Therefore, early detection and effective prevention and treatment of sarcopenic obesity holds great social significance.

Machine learning (ML) has emerged as an effective computer-aided method for data mining and analysis and is widely used as a predictive tool in different engineering and medical environments (17, 18). The principle of ML involves utilizing different algorithms to learn patterns from large and complex data in order to make predictions about unknown samples and reveal hidden predictive risk factors (19). Some studies have shown that the prediction accuracy of ML is better than that of traditional statistical methods (20, 21).

Our study aimed to identify the optimal model for predicting the occurrence of SO based on various clinical features by comparing different machine learning models. In addition, we sought to elucidate the significance of different features influencing SO, providing valuable insights for the early identification of sarcopenic obesity patients in clinical practice.

## Methods

### Study design

This is a cross-sectional study on SO in older adults. We retrospectively collected data from 386 older adult patients at the Affiliated Hospital of Qingdao University between November 2023 and May 2024. I Informed consent was obtained from all participants and/or their legal guardian(s).

Diagnostic criteria: according to the Asian Working Group for Sarcopenia (AWGS) criteria, sarcopenia is diagnosed based on two key factors: decreased muscle mass (skeletal muscle mass index (SMI) < 7 kg/m^2^ for male individuals and < 5.7 kg/m^2^ for female individuals) and decreased muscle strength (grip strength < 28 kg for male individuals and <18 kg for female individuals). In addition, obesity is defined as having a body fat percentage > 25% for male individuals and > 30% for female individuals. To diagnose SO, individuals must meet the diagnosis criteria for both sarcopenia and obesity. Older individuals aged 65 years and older were included in the study. The exclusion criteria for the study were as follows: (1) Lack of major anthropometric indicators, such as height and weight, or incomplete clinical data; (2) presence of comorbid malignant tumors, acute illnesses, or severe chronic conditions affecting the heart, lungs, kidneys, or brain; (3) diseases that affect activity and function, such as severe osteoarthritis or neuromuscular diseases; and (4) individuals with mental illness or consciousness disorders who are unable to participate in the investigation and evaluation.

This study was approved by the Research Ethics Committee of the Affiliated Hospital of Qingdao University (Ethics Approval number: QYFY WZLL 28308). All our methods were performed in accordance with the relevant guidelines and regulations.

### Data acquisition

We collected general demographic characteristics, medical history, laboratory test results, and radiological data from the patients. (1) Demographic characteristics included age, sex, smoking, and alcohol consumption. (2) Past medical history included hypertension, diabetes, cardiovascular disease, and cerebrovascular disease. (3) Laboratory tests included insulin-like growth factor-1 (IGF-1), albumin, pre-albumin, white blood cell count, neutrophils, lymphocytes, and other parameters. Upon admission, the patient’s ability to perform daily activities was evaluated using the Barthel Index score. This score ranges from 0 to 100, covering 10 items: eating, transferring between the bed and wheelchair, personal hygiene, using the toilet, bathing, walking 45 meters on level ground, climbing stairs, dressing, controlling bowel movements, and controlling bladder movements.

Body composition measures: Participants were instructed to fast for more than 2 h, empty their bladder and bowels, wear only a single garment, and sit quietly for 5 min before the test. The InBody S10 Multi-Frequency Bioelectrical Impedance Analyzer (Korea) was used to analyze the composition characteristics of different parts of the human body. Whole-body skeletal muscle mass (SMM) and appendicular skeletal muscle mass (ASM) were measured, and the limb skeletal muscle index (SMI), was calculated by dividing ASM by height squared. The InBody S10 also provided body fat percentage, visceral fat area, and bone mineral mass.

Muscle strength: muscle strength in the dominant hand was determined by taking three measurements using a handheld force gauge; only the maximum value was reported. During the assessment, the patient stood upright, supported their elbow, relaxed their shoulder, and maintained their forearm at a 90° angle to their arm.

Calf circumference: It was measured twice on each side at the largest area of the calf in the standing position. Care was taken to avoid compressing the subcutaneous tissue. The average of these measurements was then calculated.

### Statistical methods

We used SPSS (version 27.0), Python (version 3.9.16), and Scikit-learn (version 1.3.1) to analyze the data. Categorical variables were evaluated using the chi-squared test, with results presented as percentages. Continuous variables that followed a normal distribution were represented as mean ± standard deviation and analyzed using a *t*-test. Data that did not follow a normal distribution were expressed in quartiles and assessed using a non-parametric rank-sum test. A *p* < 0.05 (two-sided) was considered statistically significant.

### Selection of candidate and predictor variables

First, we performed a univariate analysis of the variables, then incorporated meaningful variables into a multivariate logistic regression model for variable screening. The results showed that BMI, grip strength, calf circumference, and Barthel Index scores were independent factors for SO. Then, the filtered predictors were input into five machine learning models for concurrent training and testing.

We selected five models for this task, namely random forest (RF), naive Bayes (NB), Light Gradient Boosting Machine (LightGBM), k-nearest neighbor algorithm (KNN), and eXtreme Gradient Boosting (XGBoost). These models were used to construct and validate a predictive model for SO. We compared the accuracy, AUC values, sensitivity, and specificity of the different models to determine the optimal prediction model for SO prevalence.

### Machine learning models

In this study, five different machine learning models were used for training and testing, namely RF, NB, KNN, LightGBM, and XGBoost.

RF is an ensemble learning method that constructs multiple decision trees based on random samples and random features to improve model accuracy.

Light GBM is an efficient gradient boosting framework that improves training speed and reduces memory consumption by optimizing decision tree construction and splitting strategies. It is often used to solve classification and regression problems.

NB is a classification method based on the assumption of independence of the Bayesian theorem and feature conditions (22). Its algorithm calculates the probabilities of various independent indicators, making it a method of probabilistic analysis.

KNN is an algorithm that predicts classification problems based on the distance between input data points and points in the training dataset.

XGBoost is an optimized gradient boosting algorithm used to improve model prediction performance by iteratively training multiple decision trees, with each tree focused on correcting the errors of the previous tree.

### Selection of machine learning models

The dataset was divided into a training and validation set in an 8:2 ratio, with 80% used for model training and 20% for validating model performance. Internal five-fold cross-validation was used to identify the most suitable hyperparameters for each model, which were then applied to each model to improve accuracy.

Evaluation metrics, including AUC and accuracy, were used to evaluate the performance of each model. The SHAP method was used to display the weight of the importance of each variable to understand their relative importance within the model.

## Results

### Clinical characteristics

Table 1 provides a comparison of baseline features between the training and test sets of the data. No significant differences were observed between the training and test sets for most features. As shown in Table 2, BMI (23.68 ± 2.68, *p* < 0.001), grip strength (17.59 ± 6.76, *p* < 0.001), and IGF-1 (75.08 ± 30.01, *p* < 0.001) were significantly lower in the SO group than in the NSO group. The age of the individuals in the NSO group was also significantly lower than that in the SO group (*p* < 0.001). The data suggest that body fat increases with age, while muscle strength decreases in older adults. Table 3 illustrates a comparison of common performance metrics across different machine learning prediction models. Based on these results, we chose the RF model as our conclusive prediction model.

**Table 1:** Baseline data for the test and training sets.

Group	Training data (*n* = 308)	Test data (*n* = 78)	*P*-value
Sex (*n*)			
Female	144 (46.8%)	37 (47.4%)	0.914
Male	162 (52.6%)	41 (52.6%)	
Hypertension (*n*)			
Yes	192 (62.3%)	51 (65.4%)	0.619
No	116 (37.7%)	27 (34.6%)	
Diabetes (*n*)			
Yes	140 (45.5%)	50 (64.1%)	0.15
No	168 (54.5%)	28 (35.9%)	
Smoking (*n*)			
Yes	30 (9.7%)	10 (12.8%)	0.425
No	278 (90.3%)	68 (87.2%)	
Drinking (*n*)			
Yes	28 (9.1%)	8 (10.3%)	0.84
No	280 (90.9%)	70 (89.7%)	
Age (year)	73 (70,79)	72 (69,78)	0.109
Weight (kg)	69.34 ± 10.94	69.19 ± 10.76	0.913
Height (cm)	161.93 ± 8.47	162.55 ± 8.89	0.58
BMI (kg/m^2^)	26.43 ± 3.69	26.12 ± 2.98	0.446
Grip strength (kg)	22.91 ± 8.39	23.94 ± 10.23	0.359
Calf circumference (cm)	35.18 ± 2.90	35.24 ± 2.90	0.873
Barthel Index score	100 (90,100)	100 (95,100)	0.753
Triglycerides (mmol/L)	1.12 (0.83,1.53)	1.07 (0.71,1.36)	0.094
Glucose (mmol/L)	5.11 (4.59,6.14)	4.75 (4.41,5.64)	0.016
Diastolic pressure (mmHg)	72.93 ± 11.06	73.17 ± 8.68	0.862
IGF-1 (ng/ml)	93.81 ± 31.91	93.63 ± 33.83	0.966
Albumin (g/L)	38.44 ± 13.90	37.94 ± 2.90	0.564
Total cholesterol (mmol/L)	4.50 ± 1.12	4.56 ± 1.27	0.695
TyG index	8.52 ± 0.64	8.31 ± 0.55	0.004*

BMI, body mass index; TyG index, the triglyceride–glucose index, was calculated as ln [triglyceride (mg/dL) × fasting plasma glucose (mg/dL)]/2. *p < 0.05.

**Table 2:** Univariate analysis of the clinical characteristics of the population in the training cohort.

Group	SO	NSO	*P*-value
Sex (*n*)			
Female	24 (16.7%)	120 (83.3%)	0.733
Male	25 (15.2%)	139 (84.8%)	
Hypertension (*n*)			
Yes	32 (10.4%)	161 (52.3%)	0.067
No	17 (5.5%)	98 (31.8%)	
Diabetes (*n*)			
Yes	24 (7.8%)	116 (37.7%)	0.589
No	25 (8.1%)	143 (46.4%)	
Smoking (*n*)			
Yes	4 (1.3%)	26 (8.5%)	0.685
No	45 (14.6%)	233 (75.6%)	
Drinking (*n*)			
Yes	4 (1.3%)	25 (8.1%)	0.881
No	45 (14.6%)	234 (76%)	
Age (years)	79 (72.5, 83.5)	72 (69, 77)	<0.001***
Barthel index score	95 (75, 100)	100 (95, 100)	<0.001***
Triglycerides (mmol/L)	1.08 (0.8, 1.43)	1.11 (0.8, 1.51)	0.427
BMI (kg/m^2^)	23.68 ± 2.68	26.96 ± 3.63	<0.001***
Grip strength (kg)	17.59 ± 6.76	23.92 ± 8.30	<0.001***
Calf circumference (cm)	32.11 ± 2.45	35.76 ± 2.61	<0.001***
Diastolic pressure (mmHg)	72.24 ± 10.26	73.06 ± 11.22	0.616
IGF-1 (ng/mL)	75.08 ± 30.01	97.35 ± 31.06	<0.001***
Albumin (g/L)	35.64 ± 4.61	38.97 ± 14.97	0.004**
Total cholesterol (mmol/L)	4.71 ± 1.40	4.46 ± 1.06	0.15

SO, Sarcopenic Obesity; NSO, Non-Sarcopenic Obesity. *p < 0.05, **p < 0.01, ***p < 0.001.

**Table 3:** Comparison of performance metrics for the different machine learning models.

Model	Accuracy	AUC	Sensitivity	Specificity
RF	86.8%	83.9%	45.5%	93.9%
LGBM	86.8%	80.8%	63.6%	87.7%
KNN	88.2%	79.4%	63.6%	92.3%
NB	73.7%	81.5%	72.7%	73.9%
XGboost	80.3%	79.8%	72.7%	81.5%

### Selection of predictors

We explored factors influencing the occurrence of SO through univariate and multivariate logistic regression analyses of the included clinical data. Based on the results, we selected the following variables for inclusion in the predictive model: BMI, grip strength, calf circumference, and Barthel Index score.

### Multiple machine learning model performance

**Figure 1:**
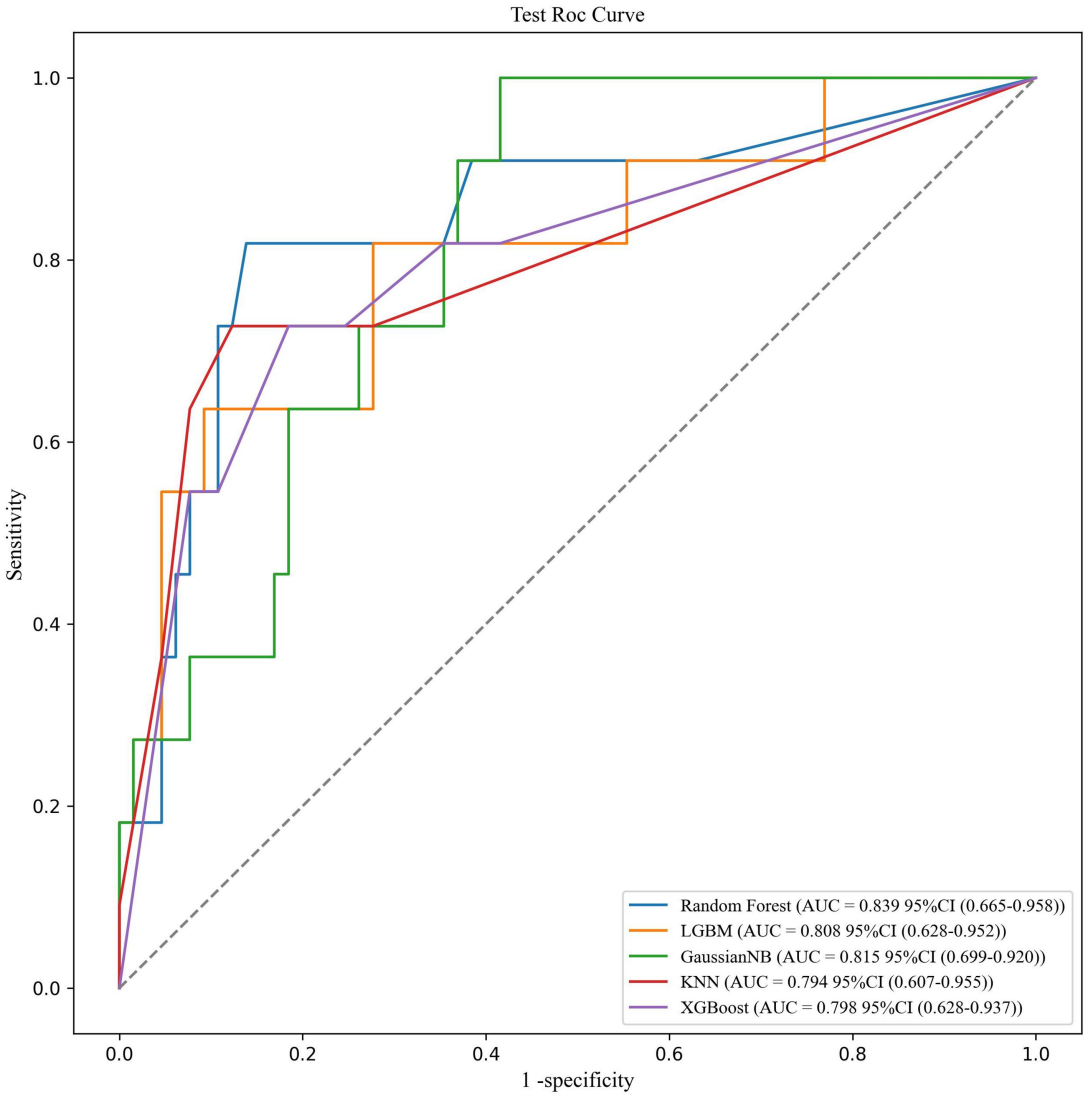
ROC curve analysis of the five machine learning alorithms for predicting SO patients in the test data.

**Figure 2:**
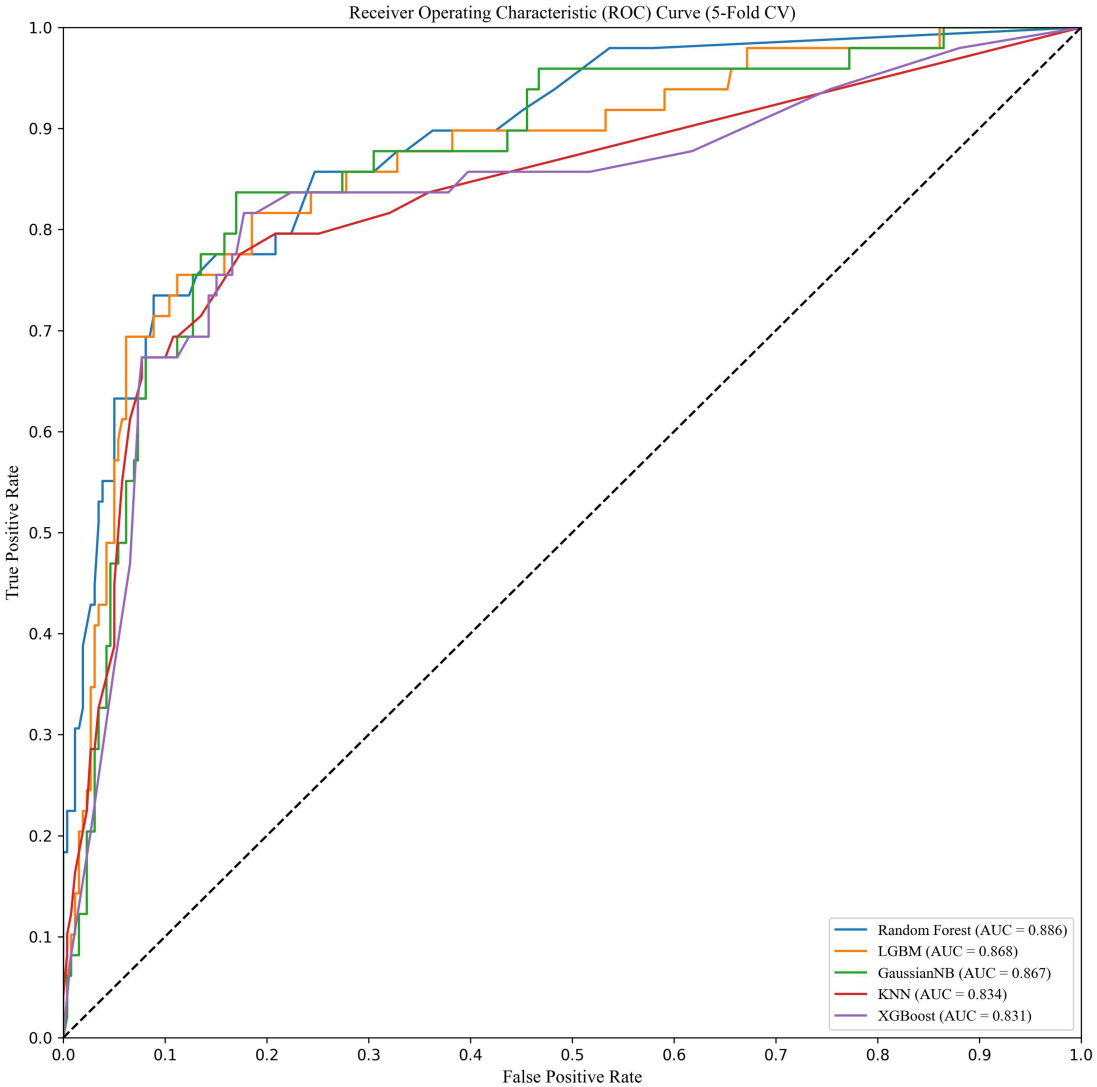
5% fold verification of internal drawings.

We trained and tested the models using the previously selected features. The results showed that the RF model was the optimal model, with an AUC value of 0.839 (Figure 1). We compared common performance metrics across the different machine learning predictive models. Based on these results, we selected the RF model as our prediction model. Figure 2 shows an internal diagram of the machine learning models after five-fold cross-validation.

### Variable importance and variable interpretation

**Figure 3:**
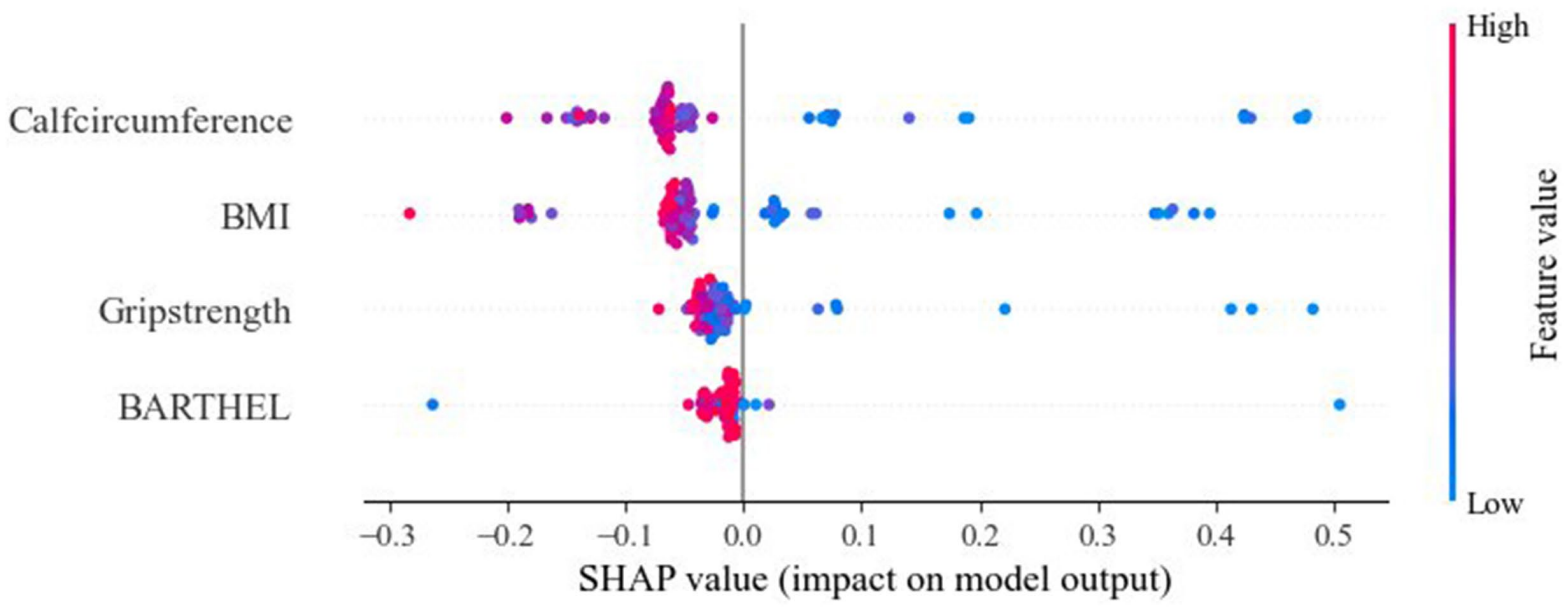
SHAP analyses of the RF model for SO patients.

We visualized the impact of the predictor variables on the results using SHAP plots. Specifically, the transverse SHAP values indicate the direction of each feature’s contribution to the predicted outcome (positive values increase the risk of sarcopenic obesity, while negative values decrease the risk). The color reflects the size of the eigenvalues: blue indicates low values, where an increase in the eigenvalues decreases the SHAP value, and red indicates high values, where an increase in the eigenvalue leads to an increase in the SHAP value. For example, the blue dots (low values) for calf circumference are concentrated in the SHAP>0 region, indicating that smaller calf circumference significantly increases the risk of SO. The SHAP analysis results showed the importance of BMI, grip strength, calf circumference, and Barthel Index score in predicting SO, with calf circumference being the most significant feature. Among these, lower calf circumference and BMI were associated with a higher likelihood of SO (Figure 3).

## Discussion

Currently, the diagnostic criteria for SO are not clear. Although there are many studies investigating the prevalence of SO, the results vary widely, with reported prevalence rates ranging from 0% to more than 41% (23), which may be influenced by different factors such as year, geographic region, study setting, and diagnostic criteria for sarcopenia (24). In this study, the prevalence of SO was found to be 15.8%, with a higher proportion in women than in men (16.7 vs. 15.2%, Table 2). It is estimated that by 2050, the proportion of the global older adult population aged 65 and above will rise to 21% (25). With the accelerated progression of aging, the health problems among older adults have gradually attracted social attention. A Taiwanese study showed that SO is associated with the highest risk of metabolic syndrome (26). Several multi-regional cross-sectional studies in South Korea have shown that older adults with SO have a higher risk of cardiovascular disease, including hyperglycemia, hypertension, hyperlipidemia, insulin resistance, and decreased cardiopulmonary function (27–29). Therefore, the individual harm and social risk brought by SO should not be underestimated. We should prioritize screening for SO in older adults and implement appropriate intervention measures in advance to reduce the occurrence of various adverse outcomes, improve quality of life, and promote healthy aging (24).

With the development of machine learning, the RF model has become a superior method for building relevant medical prediction models. A previous study by Huang et al. (30) has shown that RF models can improve the predictive power of acute respiratory distress syndrome. The random forest model developed in this study demonstrated strong discriminatory performance (AUC value of 0.839) and high specificity (93.9%) but relatively low sensitivity (45.5%). These characteristics suggest that the model is better suited as a screening tool for high-risk populations within an “initial screening followed by confirmation” diagnostic workflow, rather than serving as a standalone diagnostic criterion. Specifically, (1) high specificity minimizes false positives (avoiding unnecessary interventions for NSO individuals); (2) the sensitivity limitation could be addressed by adjusting prediction thresholds; and (3) considering clinical practicality, we recommend applying the model for initial SO risk screening in community-dwelling older adults (with positive cases referred to specialists) and for participant stratification in clinical research. This approach aligns with the stepwise diagnostic process recommended by the latest European Working Group on Sarcopenia in Older People (EWGSOP) guidelines. Future research should focus on developing a decision support system incorporating this model, enabling healthcare institutions to customize risk thresholds based on their resource availability.

This study included data from 386 older adults aged 65 years and above, analyzed the basic characteristics and clinical trial data, and applied a series of different machine learning algorithms to identify the best predictive model for predicting SO in this population. The results of this study are represented by the area under the receiver operating characteristic curve, with the RF model being the most prominent, with an AUC value of 0.839. In this study, four of the most critical predictors were identified from a large number of variables, namely BMI, grip strength, calf circumference, and Barthel Index score. The RF model predicted the prevalence of SO with an AUC value of 0.839, demonstrating a high predictive performance. Our proposed prediction metrics are readily available in community and outpatient screening settings, enabling rapid prediction of clinical outcomes.

BMI is a clinical indicator used to quantify the degree of obesity by assessing an individual’s weight status through the ratio of weight to height. Body composition in older adults can change significantly with age, characterized by a progressive decline in muscle mass accompanied by a gradual increase and redistribution of fat, such as intramuscular fat infiltration and abdominal fat accumulation, which results in an increased BMI. Although BMI reflects overall changes in body composition and does not capture specific fat distribution or muscle mass, it does indicate changes in body fat mass. In some cases, obese individuals have a higher BMI, which may imply an increase in body fat. In a cohort study in Italy, BMI was found to have similar diagnostic accuracy in identifying MetS as WC, W/H, or body mass fat index (BMI x fat mass % impedance x WC) when comparing different obesity indices and body composition (31). Results from a cross-sectional study in Spain suggested that patients diagnosed with sarcopenia tend to have more severe functional impairment and lower BMI compared to those without sarcopenia (32). A Dutch study found a negative correlation between sarcopenia and BMI (33). This aligns with the findings of this study, which showed that the BMI of the SO group was significantly lower than that of the NSO group (Table 2). A study found that obesity defined by BMI is associated with increased risks of cardiovascular disease and mortality, further supporting the evidence of BMI as a risk predictor for SO (34). Older adults often cannot tolerate various tests due to decreased physical strength and restricted mobility, making BMI, which can be calculated from height and weight, a useful tool for monitoring long-term SO risk factors in the community. This is particularly helpful in identifying the possibility of muscle mass reduction alongside weight gain. For instance, if a person is gaining weight but their BMI increases beyond the normal range, it may create concerns about a potential decrease in muscle mass.

Low grip strength is a clinical sign of low individual mobility. A South Korean study showed that grip strength in older women was positively correlated with the identification of SO. It showed that absolute grip strength is the most important factor in predicting the possibility of muscular dystrophy in older adults, further validating grip strength as a highly predictive functional indicator of SO (35, 36). The results of this study showed that, compared to the NSO group, grip strength was significantly lower in the SO group (Table 2), which is consistent with previous results. Low grip strength may be related to increased levels of pro-inflammatory cytokines, which reduce muscle strength and develop into SO (37). In addition, because the grip strength test is simple and easy to perform, it has been recommended by a number of relevant international guidelines as a preferred indicator for the evaluation and diagnosis of muscle attenuation syndrome Supplementary Table S1.

Calf circumference is a representative anthropometric indicator used in sarcopenia screening, and the definition of sarcopenia differs between the EWGSOP and EWGSOP2 and the Asian Working Group for Sarcopenia (AWGS). At present, bioelectrical impedance analysis (BIA) and dual-energy X-ray absorptiometry (DXA) are commonly used in clinical practice to measure body composition and assess sarcopenia. However, they both have specific limitations, for example, BIA is unsuitable for patients with implantable pacemakers and DXA can be costly and is often unavailable in community settings. Previous research data have shown a positive correlation between calf circumference and DXA-measured (ASM) and ASM/height^2^ (38–43), suggesting that calf circumference can serve as a screening tool for muscle mass. This is similar to the findings of our study. In addition, calf circumference measurement is simple, feasible, reproducible, and efficient, making it well-suited for use in community settings Supplementary Table S2.

The Barthel Index objectively evaluates a patient’s independent daily living ability by assessing the function of their nerves, muscles, and bones. It helps understand a patient’s daily healthcare needs and provides better quality, targeted healthcare guidance. In this study, the Barthel Index score of the SO group was significantly lower than that of the NSO group (Table 2), indicating that people with low musculoskeletal function have a higher likelihood of developing SO. In a study of women with subacute hip fractures, women with sarcopenia had lower Barthel Index scores at the end of inpatient rehabilitation compared to those without sarcopenia (44). Similarly, a Spanish study of 334 hospitalized older patients found that muscle mass is negatively correlated with poor nutritional status and impaired ability to perform basic daily activities (32). These findings further support the results of this study. Patients with different Barthel scores receive tailored healthcare based on their level of need, with personalized interventions designed to address their physical and mental requirements. The Barthel Index score is a convenient and efficient tool in community and hospital settings, facilitating early detection and follow-up of older patients with muscular dystrophy.

The strength of this study lies in the comparison of the prevalence of SO predicted by different ML models. Internal validation performance and model comparisons further proved that the RF model has good predictive value for SO prevalence. At the same time, we identified four predictors related to SO: BMI, grip strength, calf circumference, and Barthel Index score. The measurement of these four indicators is simple, and the requirements for testers and collaborators are low. In today’s context of rapid population aging, these measurements can be the first choice for large-scale population surveys and long-term community health monitoring. Although BMI, grip strength, calf circumference, and Barthel Index scores have been recognized in the diagnosis of myotonia, research on their role in SO is still insufficient. At present, since the diagnostic criteria of SO are not unified, research on SO mostly focuses on its pathogenesis and prevalence. Through this study, we emphasize the importance of BMI, grip strength, calf circumference, and Barthel Index score as early predictors of SO, thereby enriching the existing knowledge on SO and providing direction for further research.

## Conclusion

In summary, we developed a predictive model based on the RF model that integrates four easily and quickly obtainable predictors. The model demonstrates reliable predictive power for SO prevalence and can be used to predict SO in older adults.

### Limitations

This study has several limitations. First, as a cross-sectional analysis, it could not establish causal relationships between SO and the associated factors. Second, due to the relatively small sample size (total *n* = 386, SO cases = 61) and low proportion of positive cases (15.8%), the RF model might have carried some risk of overfitting, although we mitigated this issue through rigorous five-fold cross-validation and feature selection (limiting the number of variables to four). In addition, the current sample size may be insufficient to support stable calibration curves and decision curve analysis (DCA). Therefore, this study serves as a preliminary exploratory analysis aimed at providing proof of concept for machine learning-based SO prediction in older adults. Future research should include external validation using multicenter, larger-scale cohorts and prospective studies to enhance the reliability and robustness of the findings.

## Data Availability

The raw data supporting the conclusions of this article will be made available by the authors, without undue reservation.
