# Supplementary material for: Construction of a prediction model for sarcopenic obesity based on machine learning
Source: Front Public Health. 2025 Jun 27;13:1576338. doi: 10.3389/fpubh.2025.1576338 (PMC12245774; doi:10.3389/fpubh.2025.1576338)
Supplement: Supplementary file 1 [file Table_1.docx]

Table S1. Multivariate logistic regression analysis of SO

| Group | SO | NSO | *p-*value |
| --- | --- | --- | --- |
| barthel index score | 95(75,100) | 100(95,100) | ＜0.02 |
| BMI(kg/m^2^) | 23.68±2.68 | 26.96±3.63 | ＜0.001*** |
| Grip strength(kg) | 17.59±6.76 | 23.92±8.30 | ＜0.001*** |
| Calfcir cumference(cm) | 32.11±2.45 | 35.76±2.61 | ＜0.001*** |

Note:SO= Sarcopenic Obesity; NSO= Non-Sarcopenic Obesity.

*p<0.05,**p<0.01,***p<0.001.
