# Supplementary material for: Construction of a prediction model for sarcopenic obesity based on machine learning
Source: Front Public Health. 2025 Jun 27;13:1576338. doi: 10.3389/fpubh.2025.1576338 (PMC12245774; doi:10.3389/fpubh.2025.1576338)
Supplement: Supplementary file 2 [file Table_2.docx]

Table S2. Collinearity assessment between BMI and calf circumference

| Statistical Metric | BMI | Calf Circumference |
| --- | --- | --- |
| Variance Inflation Factor (VIF) | 1.428 | 1.428 |
| Tolerance | 0.700 | 0.700 |
